# A Potent Inhibitor of Human Cytomegalovirus Infection Works Post-Entry Specifically in Differentiating Myelo-Monocytic Cells

**DOI:** 10.3390/pathogens15050520

**Published:** 2026-05-12

**Authors:** Matthew J. Murray, Alexander Hargreaves, Eleanor Bradley, Qian Lee, Yanjing Zhang, Nina Reuter, Marco Thomas, Matthew B. Reeves

**Affiliations:** 1 Institute of Infection, Immunity & Transplantation, Royal Free Campus, University College London (UCL), London NW3 2PP, UK; alexander.hargreaves.23@ucl.ac.uk (A.H.); e.bradley24@imperial.ac.uk (E.B.); qian.lee.17@ucl.ac.uk (Q.L.); yanjing.zkg@gmail.com (Y.Z.); 2 Novo Nordisk Foundation Center for Protein Research, Department of Molecular and Cellular Medicine, University of Copenhagen, DK-2200 Copenhagen, Denmark; 3 Danish Cancer Institute, Danish Cancer Society, DK-2100 Copenhagen, Denmark; 4 Harald zur Hausen Institute of Virology, University Hospital Erlangen, Friedrich-Alexander-Universität Erlangen-Nürnberg (FAU), 91054 Erlangen, Germanymarco.thomas@uk-erlangen.de (M.T.)

**Keywords:** anti-virals, monocytes, human cytomegalovirus

## Abstract

Human cytomegalovirus (HCMV) remains an important medical problem in multiple patient settings despite the availability of antivirals. In part, this is linked to resistance, cost and restrictions on use in several patient settings. More generally, it remains attractive to increase our arsenal of anti-viral approaches to target HCMV. We previously characterized a potent inhibitor of HCMV infection, DIDS, that displays cysteine reactivity, allowing it to bind virions and neutralize HCMV infection of fibroblasts. We now show that DIDS is inhibitory to cell-free and cell-associated infection of multiple cell types, including cells of the haematopoietic lineage—cells important for latency and dissemination. Consistent with this broad activity, DIDS partially inhibited gB (but not SARS-CoV-2 spike) fusion activity. Intriguingly, further characterization of DIDS activity in myeloid cells revealed that, unlike in all other cell types, DIDS blocked a post-entry event in CD14+ monocytes and also dendritic cell derivatives. Despite viral entry, entry was largely silent, with a failure to activate innate immunity and cell survival pathways known to be activated by HCMV. In contrast, HCMV infection was observed to activate host miRNA expression in CD14+ cells, suggesting a DIDS-insensitive viral function was responsible or, alternatively, that host miRNA expression is a potential anti-viral response to viral internalization. Thus, we report the further characterization of a broad-acting inhibitor of HCMV infection, which may also prove a useful tool to study unique events for the infection of monocytic cells by HCMV—a cell type that is crucial for HCMV dissemination and pathogenesis in vivo.

## 1. Introduction

Human cytomegalovirus (HCMV) is an important medical pathogen responsible for disease in multiple patient populations [1,2]. The pathogenesis associated with HCMV is diverse, with most organs susceptible to infection. This is, in part, due to the broad cell tropism of HCMV for differentiated cell types, including fibroblasts, epithelial, neuronal and endothelial cells [3,4]. Furthermore, the capacity of HCMV to infect both undifferentiated and differentiated myeloid cells is critical for latency, reactivation and dissemination in the host [5,6].

The broad cellular tropism of HCMV is linked to the expression of multiple glycoprotein complexes that are presented on the HCMV virion, which facilitate binding to various cell surface receptors in a cell type-specific manner [3]. These interactions’ outcomes are diverse: HCMV can infect cells via classic fusion at the plasma membrane (PM) or via the endocytic and macropinocytotic pathways [7,8,9,10]. Typically, clinical isolates that have been passaged extensively in the laboratory have displayed a restricted tropism for mainly fibroblast-like cells and generally infect via fusion at the PM, although the utilization of other pathways in fibroblasts has been reported [11,12]. Entry events are driven by both glycoprotein B (gB)—which is the primary fusogen—and also gH/gL containing complexes that are essential for both the fusogenic activity observed with gB, as well as the broad cellular tropism also imparted by gH/gL containing complexes [3,8,13,14,15]. In contrast, non-fibroblast cells are generally more resistant to infection with laboratory-adapted viruses, which is linked to the absence of the pentamer (PC) complex comprising gH/gL/UL128/UL130/UL131 [16,17,18,19,20,21]. This complex plays a key role in the utilization of endocytic pathways by the virus in these cells, including allowing virion escape from the endosome in epithelial cells [22] as well as prevent the trafficking of the virion into recycling endosomes in monocyte infection [23]. Again, gB is important for infection of these cell types, where it drives ‘fusion from within’ to promote capsid escape from the endosomal compartment [24].

Targeting HCMV entry via biological and pharmacological approaches is considered an important strategy for the prevention of HCMV disease. The demonstration that inhibitors of host cell ion channel activity could limit infection with a number of different viruses [25,26,27] led us originally to test for a role in HCMV infection [28]. In a screen of ion channel inhibitors, we previously identified DIDS as a potent inhibitor of HCMV infection of fibroblasts and epithelial cells. Further characterization suggested DIDS inhibited HCMV infection via a mechanism distinct from DIDS’ documented activity against chloride ion channels [28,29]. Specifically, we reported DIDS bound to HCMV via cysteine reactivity and that this was sufficient to prevent virus binding and entry into fibroblasts. A failure to activate ISG expression upon infection of HFFs with HCMV in the presence of DIDS led us to speculate that it prevented virus binding at the plasma membrane with phenotypic similarity to heparin [30] although via a different mechanism [28].

Here we report on the further characterization of DIDS’ anti-viral activity and now demonstrate DIDS potently blocks both cell-free and cell-associated viral infection in permissive cells. Further characterization of cell-free infection shows that DIDS blocks viral entry of multiple permissive cell types and also entry into CD34+ haematopoietic cells—a major site of HCMV latency [5,31,32,33,34]. We go onto show that DIDS partially blocks the fusogenic activity of gB (but not the SARS-CoV-2 Spike protein), suggesting that inhibiting aspects of gB function contributes to these phenotypes. We also observe that DIDS blocks infection of THP1 myelomonocytic cells, CD14+ monocytes and dendritic cells (DCs), but, surprisingly, this was observed to be post-entry, unlike in all other cell types tested. Furthermore, it was evident that viral genomes persisted in the infected THP1 and CD14+ monocytes but they were likely non-functional, being both transcriptionally silent for latent gene expression and unresponsive to stimuli for reactivation. Intriguingly, despite not preventing entry, DIDS still diminished the activation of multiple innate immune signatures but had no effect on the rapid activation of host miRNA expression we have previously observed upon viral infection of myeloid cells. Thus, we provide further characterization of an anti-viral compound, DIDS, with the potential to also be utilized as a tool to probe for differences in the infection of multiple cell types during HCMV infection.

## 2. Materials and Methods

### 2.1. Cells and Viruses 

Adult retinal pigment epithelial cell line ARPE-19 (CRL-2302), monocyte cell line THP-1 cells (TIB-202) and primary human fetal foreskin fibroblasts (HFFs, SCRC-1041) were purchased from ATCC. Human glioblastoma astrocytoma cells (U373) were a kind gift from John Sinclair, University of Cambridge. HFF-TET cells were a kind gift from Richard Stanton, Cardiff University. All cells were grown at 37 °C with 5% CO_2._ HFF, U373 and HFF-TET cells were grown in high-glucose Dulbecco’s modified Eagle medium (DMEM, ThermoFisher, Altrincham, WA14 2DT, UK, Altrincham, WA14 2DT, UK), ARPE-19 cells were grown in DMEM:F12 (ThermoFisher, Altrincham, WA14 2DT, UK, Altrincham, WA14 2DT, UK) and THP-1 cells were grown in RPMI-1640 (ThermoFisher, Altrincham, WA14 2DT, UK, Altrincham, WA14 2DT, UK). All were supplemented with 10% fetal bovine serum (FBS, ThermoFisher, Altrincham, WA14 2DT, UK) and 1% penicillin/streptomycin (P/S).

Primary monocytes were isolated from blood donated by healthy donors. Blood was diluted 1:1 with Ca^2+^/Mg^2+^-free phosphate-buffered saline (PBS) prior to layering over Histopaque 1077 (Sigma-Aldrich, Gillingham, SP8 4XT, UK). Blood was fractionated by centrifugation (20 min, 850 g, room temperature, no brake). Peripheral blood mononuclear cells (PBMCs) were removed from the interface and washed with Ca^2+^/Mg^2+^-free PBS. CD14+ monocytes were then isolated by magnetic-activated cell sorting (MACS) as per the manufacturer’s instructions. Briefly, CD14+ monocytes were labelled with CD14 microbeads (Miltenyi Biotech, Bisley, GU24 9DR, UK) prior to positive selection on LS+ columns (Miltenyi Biotech, Bisley, GU24 9DR, UK). Once eluted from the column, CD14+ cells were seeded in Ca^2+^/Mg^2+^-containing PBS into tissue culture plates. After 1 h, PBS was removed and replaced with RPMI-1640 supplemented with 10% FBS and 1% P/S. Isolation of primary dendritic cells followed the same initial procedure for isolation of PBMC and was performed on the CD14^neg^ fraction collected after CD14+ monocyte isolation using a specific myeloid dendritic cell isolation kit (Miltenyi Biotech, Bisley, GU24 9DR, UK). Briefly, cells are incubated with a non-DC depletion cocktail and then biotin microbeads and purified on an LD column (Miltenyi Biotech, Bisley, GU24 9DR, UK) with unbound cells representing a population enriched for CD141 and CD1c expressing DCs.

Purchased CD34+ cells (Lonza, Slough, SL1 4DX, UK) were resuscitated and maintained in X-VIVO 15 serum-free media for 48 h prior to use in HCMV infections. No long-term culture or expansion of CD34+ cells was performed in vitro.

The Merlin clinical virus strain and Merlin IE2-GFP virus were gifts from Richard Stanton, Cardiff University. The TB40/e strain of HCMV was a kind gift of Christian Sinzger, Ulm University. All viruses were stored at −80 °C. Merlin-IE2-GFP is a BAC recombinant HCMV strain containing RL13 and UL128-131A under the control of a tet repressor so that it grows predominantly in a cell-associated growth mode in HFFs. The virus is propagated as a cell-free virus in HFF-Tet cells to prevent mutations of UL128-131. Genetically wild-type stocks can then be used in normal HFFs to study cell-associated growth. GFP expression with IE2 kinetics allows real-time visualization of infection. The Merlin WT HCMV used in the lab has been subsequently passaged in HFFs, mutating to grow as a predominantly cell-free virus. The TB40/e virus was propagated in ARPE-19 cells with amplification prior to use in a single passage in HFFs to produce cell-free virus with myelo-tropism.

### 2.2. Viral Infection, Immune Staining and Neutralization Assays

For standard infections, viruses were incubated with cells for 3 h (unless stated otherwise) and then washed off, and fresh media was added to the cells. For single-step growth curves, cells were infected at an MOI = 1, and for multi-step growth curves and viral dissemination assays, cells were infected at an MOI = 0.05.

After infection was completed, cells were washed for 5 min with PBS, prior to the addition of ice-cold ethanol (−20 °C). Cells were then incubated at −20 °C for at least 20 min. To stain for viral IE proteins, ethanol was removed, and cells were washed in PBS for 5 min. Cells were then incubated with 1:2000 α-IE antibody (clone 6F8.2, Merck Millipore, 64293 Darmstadt, Germany) in PBS for 1 h. Cells were washed in PBS again, then incubated with 1:2000 anti-Mouse IgG-Alexa-fluor-568 nm (Life Technologies, Paisley, PA4 9RF, UK) and 1:2000 DAPI (Sigma-Aldrich, Gillingham, SP8 4XT, UK) in PBS for 1 h in the dark. Cells were washed once more with PBS, fresh PBS added, and plates stored at 4 °C prior to quantification.

For neutralization assays, HCMV was incubated with the AD-2 monoclonal antibody ITC-88 for 1 h prior to the addition of complexes to cells. Alternatively, ITC-88 was added at the time of infection or 1 hpi as indicated in the specific legend. All cells were washed, and fresh media were replaced after 3 h and immune-stained at 24 hpi and quantified as described below.

A Hermes WiScan (IDEA Bio-Medical, Rehovot, 7670502, Israel) automated microscope was used to capture 50% coverage/well with images processed using MetaMorph microscopy automation and image analysis software (Molecular Devices, CAT#: STEM-LE-0521-LC). Relative infection was calculated from the analyzed microscopy images as N^o^ IE-positive DAPI-positive cells/total N^o^ DAPI-positive cells. Infection was then standardized by setting the control to 1, and infections were expressed relative to the control.

### 2.3. Infection Assays in Sites of Latency and Reactivation

250,000 CD14+ monocytes were infected at MOI = 5 with HCMV strain TB40/E. One day post-infection, cells were washed with PBS and media replenished, with 1000 U/mL interleukin-4 (IL-4, Peprotech, Rocky Hill, NJ, USA) and granulocyte/macrophage-colony stimulating factor (GM-CSF, Peprotech, Rocky Hill, NJ, USA) added after a further 2 days to induce differentiation into dendritic cells where required. Cells were maintained for an additional 6 days, then cells were harvested for RNA or further treated with 50 ng/mL recombinant interleukin-6 (IL-6, Peprotech, Rocky Hill, NJ, USA). RNA samples were harvested from IL-6-treated cells 24 h post stimulus. RNA was then processed and analyzed by qRT-PCR as described below.

### 2.4. Inhibitors and Chemicals

The chloride channel inhibitor 4,4′-diisothiocyano-2,2′-stilbenedisulfonic acid (DIDS) was purchased from Sigma-Aldrich. NPPB was a kind gift from Jamel Mankouri (University of Leeds). Dimethyl sulfoxide (DMSO) was used as the solvent control for all experiments. The stilbene derivative, SITS (4-Acetamido-4′-isothiocyanostilbene-2,2′-disulfonic acid; Sigma-Aldrich, Gillingham, SP8 4XT, UK), was used for comparison with DIDS, which, despite a similar scaffold and charge, differs from DIDS due to a loss of a reactive isothiocyanate group. Cisplatin A (CspA) was used as described previously [35] and was used at 10 uM in all experiments. Decanoyl-RVKR-CMK was used to inhibit furin activity associated with Spike protein activation.

### 2.5. Nucleic Acid Isolation and Analysis

To isolate DNA, cells were washed in PBS for 5 min, prior to the addition of a solution of 100 mM KCl, 10 mM Tris-Hcl and 2.5 mM MgCl for 5 min. An equal volume of 10 mM Tris-HCl, 2.5 mM MgCl, 1% *v/v* Tween-20, 1% *v/v* Nonidet P-40 (Santa Cruz Biotechnology, Santa Cruz, CA, USA) and 0.4 mg/mL proteinase K (Sigma-Aldrich, Gillingham, SP8 4XT, UK) solution was added for a further 5 min. The solution was then heated for 60 min at 60 °C, followed by 10 min at 95 °C and the extracted DNA was stored at −20 °C.

Total RNA was extracted using the Qiagen RNeasy kit, using columns from Epoch Life Sciences, according to the manufacturer’s protocol. cDNA was synthesized from an equal quantity of RNA per sample using the Qiagen Quantitect Reverse Transcription kit as per the manufacturer’s (Qiagen, Manchester, M13 0BH, UK).

To harvest miRNAs, CD34+ cells or CD14+ monocytes were either mock-infected or HCMV-infected at MOI = 5, and after 6 h, small RNAs (<200 nt) were extracted using the PureLink™ miRNA Isolation Kit (two-column method, ThermoFisher, Altrincham, WA14 2DT, UK). Samples were subjected to first-strand cDNA synthesis. Briefly, equal quantities of small RNA per sample (15–50 ng) were mixed with *E. coli* poly(A) polymerase reaction buffer (1x), 100 µM ATP, 100 µM dATP, 100 µM dCTP, 100 µM dGTP, 100 µM dTTP, 1 µM universal RT primer (CAG GTC CAG TTT TTT TTT TTT TTT VN), 100U M-MuLV reverse transcriptase, and 1U *E. coli* poly (A) polymerase. Samples were incubated at 42 °C for 1 h, followed by reaction inactivation for 5 min at 90 °C. Samples were diluted 4-fold with RNase-free water.

miR-specific primers were designed using the miRprimer software (version 1) as reported previously [36]. Each qPCR reaction contained 1x PowerUp SYBR Green master mix (ThermoFisher, Altrincham, WA14 2DT, UK), 500 nM forward and reverse primer and an equal quantity of diluted cDNA. Cycling conditions were 2 min at 50 °C, 2 min at 95 °C, followed by 50 cycles of 95 °C for 10 s and 60 °C for 60 s.

Relative quantification of 18S, UL138 and total IE transcripts was performed with 1x PowerUp SYBR Green master mix with 250 nM of forward and reverse primers, using cycling conditions of 50 °C for 2 min, 95 °C for 2 min and 40 cycles of 95 °C for 15 s, 60 °C for 15 s and 72 °C for 1 min. The gene-specific primers employed were: UL138 (F, GAG CTG TAC GGG GAG TAC GA; R, AGC TGC ACT GGG AAG ACA CT), 18S (F, GTA ACC CGT TGA ACC CCA; R, CCA TCC AAT CGG TAG TAG CG) and total IE (F, GGA CCC TGA TAA TCC TGA CG; R, ATC TTT CTC GGG GTT CTC GT).

All qPCR reactions were performed and analyzed using the QuantStudio 3 (ThermoFisher, Altrincham, WA14 2DT, UK) system. All qPCR reactions were performed in technical duplicate, with biological replicates as noted in the appropriate figure legend. Alternatively, PCR products were visualized on 2% agarose gels by standard gel electrophoresis and staining with SybrSafe and visualization under UV light.

For analyses of ISG expression, specific targets CXCL10, IFIT2 and IFIT3 were analyzed as previously described [36]. For global analyses of ISG expression, the RT^2^ Profiler Qiagen^®^ custom array was used. qPCR reactions were prepared to a total volume of 25 uL using ThermoFisher, Altrincham, WA14 2DT, UK PowerUp™ SYBR™ Green as per the manufacturer’s instructions. Cycling conditions were 50° C for 2 min and 95° C for 2 min, then 40 cycles of 95° C for 15 s, 60 ° C for 0.15 s and 72° C for 1 min in the 7500 RT-PCR System thermal cycler from Applied Biosystems, Warrington, WA3 7QH, UK).

### 2.6. Dual-Split Protein (DSP) Fusion Assay

The gB and Spike protein DSP fusion assays were carried out as previously described in which the activity of the 1G2 antibody (used herein as a control) which is directed against AD5 of gB, has been described [37,38,39].

## 3. Results

### 3.1. DIDS Inhibits Lytic Infection of Multiple Cell Types

**Figure 1:**
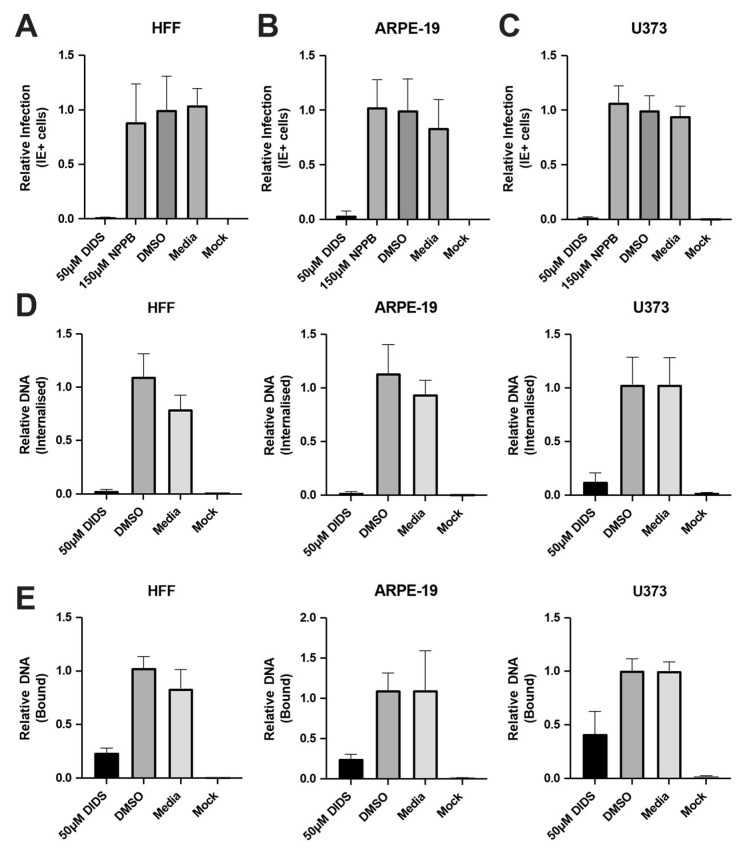
**DIDS blocks binding and entry into multiple cell types permissive for HCMV infection.** (**A**–**C**) HFFs (**A**), ARPE-19 (**B**) or U373 (**C**) cells were incubated with DIDS, NPPB, DMSO or media for 1h and then infected with HCMV and stained for IE gene expression 24 hpi, and infection was scored relative to DMSO control. (**D**,**E**) HFFs, ARPE-19 or U373 cells were pre-incubated with DIDS, DMSO, media and then infected with HCMV either at 37 °C (**D**) or 4 °C (**E**), and DNA was harvested 1 hpi and analyzed by qPCR and DNA expressed relative to DMSO control. *n* = 3 and error bars represent 1 standard deviation from the mean.

We have previously shown that DIDS is inhibitory to lytic infection of fibroblasts by limiting the binding of HCMV to the cell surface [28]. Given that HCMV has been demonstrated to utilize several routes to enter cells in a cell-type-specific manner, we first tested the activity of DIDS against other permissive cell types. HFFs, ARPE-19 and U373 astrocyte-like cells were pre-treated with DIDS and then infected with the HCMV strain, TB40/e and stained for IE gene expression. As expected, DIDS potently blocked infection of HFFs (Figure 1A). Furthermore, DIDS also inhibited lytic infection of ARPE-19 cells and U373 cells (Figure 1B,C). As observed previously [28], other chloride channel inhibitors had no effect (Figure 1A–C), arguing that the inhibitory effect was not via DIDS’ canonical pharmacological target (chloride ion channels).

The broad-spectrum activity of DIDS against the infection of multiple cell types was consistent with the identified capacity of DIDS to prevent binding of HCMV to the surface of HFFs, which, consequently, manifests as a reduction in the delivery of viral genomes to the cell. Similarly, DIDS also substantially reduced the delivery of HCMV genomes to ARPE-19 and U373 cells (Figure 1D). Furthermore, it was also clear that DIDS also limited attachment of virions to the cell in all three cell types tested (Figure 1E).

**Figure 2:**
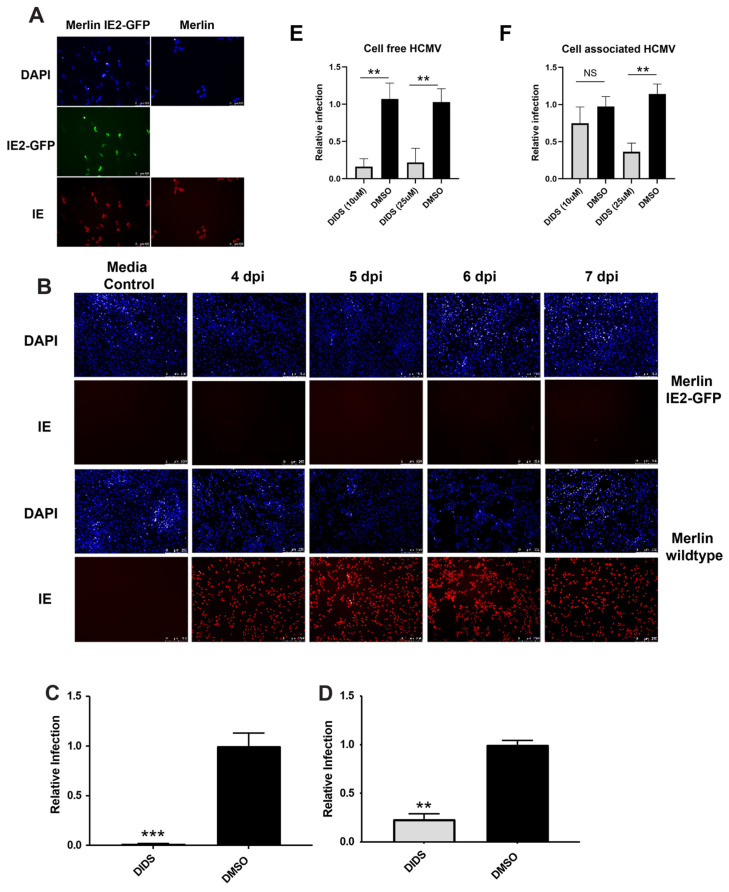
**DIDS blocks the spread of highly cell-associated HCMV.** (**A**,**B**) HFFs were infected with a highly cell-associated HCMV (Merlin IE2-GFP) or cell-free HCMV (high passage Merlin) at an MOI of 3 and left for 7 days, with supernatants collected daily 4–7 dpi. After 7 days, HFFs were stained for IE gene (red) or visualized for IE2-GFP (green) expression to confirm infection (**A**). Supernatants were used to inoculate fresh HFFs and stained for IE gene expression 24 hpi (**B**). (**C**,**D**) HFFs were infected at low MOI (0.05) with Merlin (**C**) or Merlin IE2-GFP (**D**) and then cultured for 10 days in the presence of DIDS (50 uM) or DMSO from 3 dpi. Cells were stained for IE gene expression, and infection was scored relative to a media-only control. (**E**,**F**) HFFs were infected as above, except DIDS was added at 25 uM and 10 uM (or DMSO control) at 3 pi. Infection was quantified by IE immunostaining and scoring relative to media control. *n* = 3 for panels (**C**–**F**) and error bars represent 1 standard deviation from the mean. For (**C**,**D**), the Mann–Whitney U test was performed to compare means. Means in (**E**,**F**) were compared by Kruskal–Wallis followed by Dunn multiple comparisons test. ** *p* < 0.01; *** *p* < 0.001; NS = non-significant.

Next, we investigated whether DIDS had any anti-viral activity against cell-associated HCMV replication. To do this, we employed the Merlin-IE2-GFP virus engineered to differentially express the PC depending on the cell type infected. Specifically, the virus replicates highly cell-associated in HFFs and has been used previously to characterize the anti-viral activity of a vaccine-induced antibody response [40,41]. In parallel, we employed a lab-passaged Merlin virus stock that grows predominantly as a cell-free virus. First, we performed a single-round high-MOI infection assay to confirm that the IE2-GFP virus stock being used was almost exclusively cell-associated, whereas the Merlin produced cell-free virus (Figure 2A,B). Next, we performed a low-MOI multi-round replication assay and tested whether DIDS could block infection under these conditions. The data clearly show that the cell-free spread of Merlin is substantially reduced by DIDS (Figure 2C). Likewise, the cell-to-cell spread of cell-associated Merlin IE2-GFP was also reduced (Figure 2D), although the inhibition was less overt than that observed for cell-free Merlin virus (Figure 2C). Furthermore, the greater activity of DIDS against cell-free HCMV was also supported by data demonstrating that cell-free HCMV was more potently inhibited by DIDS at a lower concentration (Figure 2E,F). That said, the data do show that DIDS is active against both cell-free and cell-associated HCMV.

### 3.2. DIDS Displays an Extended Activity Against HCMV Infection in ARPE Cells

**Figure 3:**
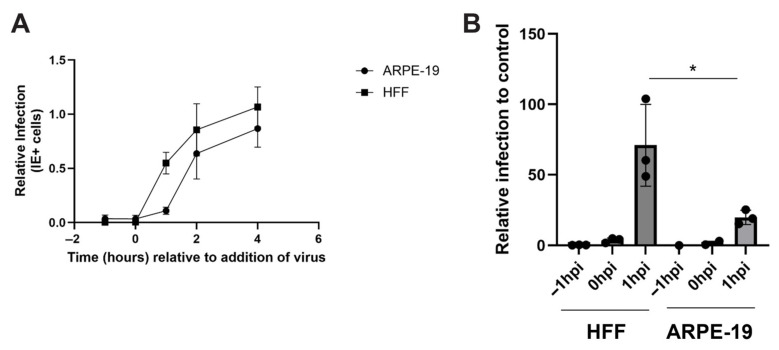
**DIDS has cell type-specific differences in time of addition studies that correlate with gB antibody phenotypes.** (**A**) HFFs or ARPE-19 cells were incubated with DIDS (50 uM) at intervals between 1 h pre-infection to 4 hpi with TB40/e and assessed for anti-viral activity against infection. Infection was scored by immunostaining for IE gene expression 24 hpi relative to the DMSO control. (**B**) HFFs or ARPE-19 cells were incubated with anti-gB neutralizing antibody ITC-88 (5 ug/mL), 1 h pre-infection to 1 hpi with HCMV. Infection was scored by immunostaining for IE gene expression 24 hpi relative to the IgG control. *n* = 3 and error bars represent 1 standard deviation from the mean. A two-way ANOVA analysis with multiple comparisons was performed. * *p* < 0.05.

During the course of the characterization of DIDS as an anti-viral, we performed time of addition studies to see how rapidly DIDS could impart anti-viral activity against HCMV infection. Cells were either pre-treated or incubated with DIDS at the time of viral infection or at times post addition of the virus (Figure 3A). The data show that DIDS was potently anti-viral against HCMV infection of both HFFs and ARPE-19 cells, even when added at the same time as the virus. This would be consistent with our previous data, suggesting DIDS blocked a viral function required for binding and/or entry rather than the requirement to inhibit a host function [28]. Notably, however, we observed that whilst the addition of DIDS post-infection meant that it no longer inhibited HFF infection, it did still have demonstrable activity against ARPE-19 infection (Figure 3A). To investigate this further, we compared this phenotype with the potent neutralizing antibody ITC-88 [42] directed against the AD-2 epitope of gB under a similar experimental set-up, since antibodies against AD-2 have been shown to inhibit HCMV infection post-binding [43,44]. Interestingly, the data suggest that ITC-88 had the same profile as DIDS in these assays—whereby it was inhibitory to HCMV infection of ARPE-19 cells post-addition of the virus for longer than that seen with HFFs (Figure 3B).

### 3.3. DIDS Partially Inhibits the Fusogenic Activity of gB but Not Spike Protein

**Figure 4:**
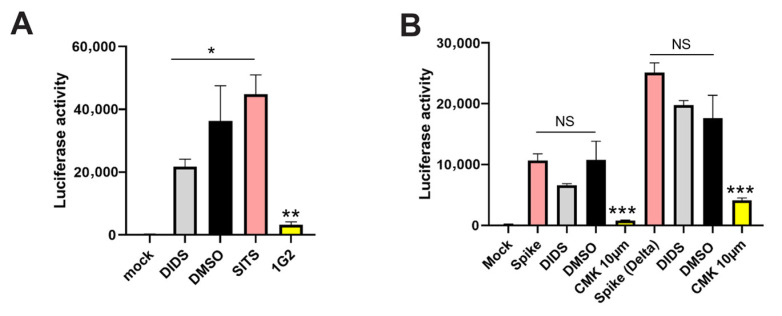
**DIDS partially inhibits gB, but not SARS-CoV-2 spike, activity in vitro.** (**A**,**B**) 293T-DSP-mix cells were transfected with plasmids encoding gB-VSVG (**A**) or CoV-2 Spike or Spike Delta variant (**B**). DSP cells were then cultured in the presence of DIDS, DMSO, SITS or 1G2 (**A**) to measure the impact on fusion activity of HCMV gB. Alternatively, DSP cells were cultured with DIDS, DMSO or a furin inhibitor (CMK) (**B**). Cell–cell fusions were quantified using the DSP assay and luciferase reporter activity. *n* = 3 and error bars represent 1 standard deviation from the mean. A Brown-Forsythe and Welch ANOVA test with Dunnett’s multiple comparisons (**A**) or a one-way ANOVA with Sidak’s multiple comparisons was performed (**B**). * *p* < 0.05; ** *p* < 0.01; *** *p* < 0.001; NS = non-significant.

Our previous study suggested that DIDS was anti-viral through its capacity to bind to virions. Given that we have now observed that DIDS had activity against cell-associated spread of HCMV, we hypothesized that DIDS activity was directed against at least one of the glycoprotein complexes important for entry and spread. To investigate this, we employed a novel in vitro fusion assay recently used to characterize a potent anti-gB antibody specific for AD-5 [38,39] and also modified to study SARS-CoV-2 Spike activity [37]. The gB-DSP assay uses luciferase as a measure of fusion once the two halves of the gB fusion machinery are brought together. As expected, the AD5 antibody (1G2) blocked gB activity. We also observed that DIDS partially blocks gB fusogenic activity by about 50% (Figure 4A). In contrast, these effects were not directed against all viral fusion proteins—DIDS had no activity against SARS-CoV-2 Spike protein under these assay conditions (Figure 4B). Taken together, these data demonstrated that DIDS was active against multiple routes of lytic infection and that potentially that was due to activity, at least in part, against gB.

### 3.4. DIDS Inhibits Myelo-Monocytic Infection at a Post-Entry Stage of Infection

**Figure 5:**
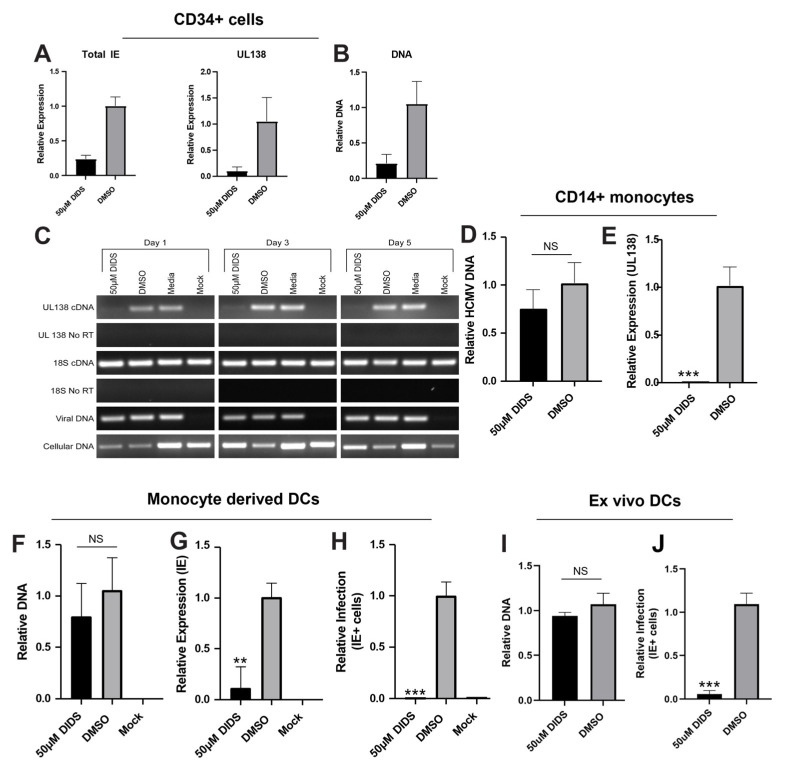
**DIDS inhibits infection of myelomonocytic cells post-entry.** (**A**,**B**) CD34+ cells were incubated with DIDS (50 uM) for 1 h prior to infection with HCMV TB40/e and then analyzed by qRT-PCR for IE and UL138 RNA expression (**A**) or by qPCR for viral DNA (**B**) 24 hpi. All data expressed relative to media control cells infected with TB40/E. (**C**–**E**) THP1 cells (**C**) and CD14+ monocytes (**D**,**E**) were pre-incubated with DIDS (50 uM) or DMSO and then infected with HCMV and analyzed for UL138 RNA expression (**C**,**E**) or viral genome carriage by qPCR (**C**,**D**). All data expressed relative to media control cells infected with TB40/E (**D**,**E**). (**F**–**H**) Monocyte-derived DCs were generated from primary CD14+ monocytes and then, following 1 h pre-treatment with DIDS (50 uM) or DMSO control, were infected with TB40/E and analyzed by qPCR for viral DNA (**F**), by qRT-PCR for IE RNA expression (**G**) or IE immunostaining (**H**). All data sets were expressed relative to a media control and HCMV infection at 24 hpi. (**I**,**J**) circulating DCs were directly isolated from peripheral blood and pre-incubated with DIDS (50 uM) or DMSO control for 1 h prior to infection and then analyzed by qPCR for viral genomes (**I**) and qRT-PCR for IE RNA expression at 24 hpi. All data expressed relative to HCMV infection in media control. *n* = 3 and error bars represent 1 standard deviation from the mean, except for C, which is a representative panel of two independent experiments. Mann–Whitney U comparison of the means was performed. ** *p* < 0.01; *** *p* < 0.001; NS = non-significant.

Our long-standing interest in HCMV latency and the myeloid lineage led us to complete our cellular characterization of DIDS activity against CD34+ haematopoietic progenitors and more differentiated myeloid cells. Again, consistent with the data for lytic infection, we observed that CD34+ cell infection was blocked by DIDS and that again this was clearly at the level of entry (Figure 5A,B). Thus, we were surprised to see that the impact of DIDS on the infection of THP1 monocytic cells and CD14+ monocytes presented with a different phenotype to all other cells tested thus far. Specifically, the data suggested that intracellular viral genomes were evident following the infection of monocytic cells (Figure 5C,D). Notably, these genomes were not transcriptionally active (Figure 5C,E). Furthermore, a similar phenotype was observed in monocyte-derived DCs (MoDCs), whereby DIDS clearly blocked IE gene expression but the block was again appearing to occur post-genome entry (Figure 5F–H). Mindful that the MoDCs phenotype could be linked to generating DCs from monocytes in vitro [45], we directly isolated circulating DCs from peripheral blood and also observed that DIDs blocked infection at a post-entry stage (Figure 5I,J). Thus, DIDS was inhibitory to infection of all cell types but in myeloid-committed cells (e.g., THP1 cells, monocytes and DCs), it displayed a differential post-entry inhibitory phenotype which we decided to investigate further.

### 3.5. The Persisting Genomes in Myelo-Monocytic Cells Do Not Trigger Innate Immune Responses and Cannot Be Reactivated

**Figure 6:**
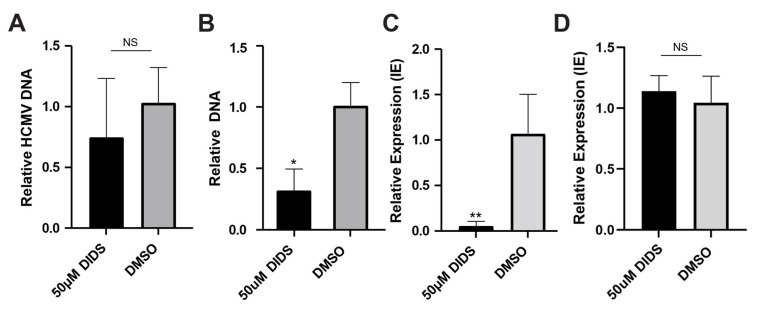
**Viral DNA persists in CD14+ monocytes but is transcriptionally inactive.** (**A**–**C**) CD14+ monocytes pre-treated with DIDS (50 uM) or DMSO control for 1 h were then infected with HCMV and analyzed at 72 hpi for viral DNA by qPCR (**A**) or were subsequently differentiated to MoDCs and analyzed for viral DNA by qPCR (**B**) or, following IL-6 stimulation to promote reactivation, analyzed for IE gene expression by qRT-qPCR. (**D**) CD14+ monocytes were infected with HCMV, differentiated to MoDCs, and then, prior to the addition of IL-6 to promote reactivation, were incubated with DIDS (50 uM) or DMSO for 1 h. 24 h post IL-6, cells were analyzed by qRT-PCR for IE gene expression. All datasets are expressed relative to the media-infected control. *n* = 3 and error bars represent 1 standard deviation from the mean. Mann–Whitney U comparison of the means was performed. * *p* < 0.05; ** *p* < 0.01; NS = non-significant.

We noted that the THP1 analyses revealed that not only did viral genomes enter the cells but they persisted during culture following infection (Figure 5C). Furthermore, the genome persistence occurred in the absence of detectable latent gene expression; thus, we investigated whether viral genomes could also persist in CD14+ primary cells. A qPCR analysis of cells infected in the presence of DIDS suggested this was also true in CD14+ cells, although a two-fold reduction was evident by 3 dpi (Figure 6A). Furthermore, differentiation of the cells to MoDCs had no dramatic impact on viral genome levels (Figure 6B), and reactivation of IE gene expression could not be induced with IL-6 (Figure 6C) if the CD14+ cells had been pre-treated with DIDS at the time of infection. However, DIDS itself was observed not to be inhibitory to HCMV reactivation if added only at the time of IL-6 addition to MoDCs (Figure 6D). DIDS was ‘inhibitory’ to HCMV reactivation only if the initial infection of the CD14+ monocytes was performed in the presence of DIDS (that is, a defect in the establishment of functional latency, logically, resulted in a defect in reactivation).

**Figure 7:**
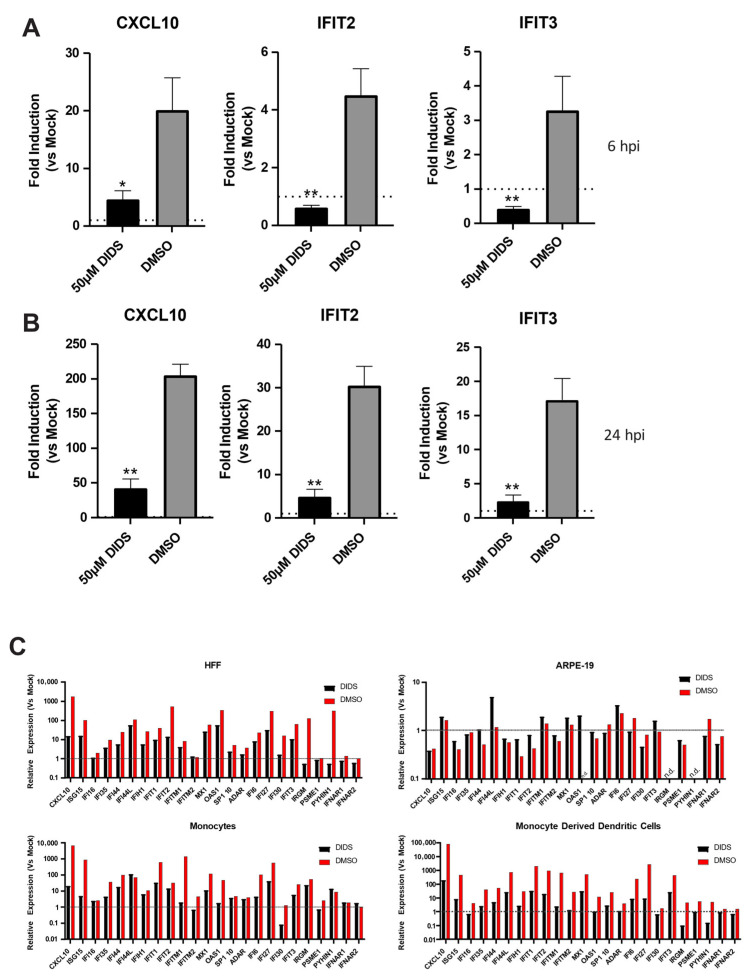
**DIDS reduces ISG induction by viral infection.** (**A**,**B**) CD14+ monocytes were pre-treated with DIDS (50 uM) or DMSO and then infected with HCMV and analyzed for ISG expression at 6 and 24 hpi by qRT-PCR. All datasets are expressed relative to infected media control. *n* = 3 and error bars represent 1 standard deviation from the mean. Dashed line represents 1 (no change) For (**A**,**B**) Mann–Whitney U comparison of the means was performed. * *p* < 0.05; ** *p* < 0.01 (**C**) An array analysis for the expression of a panel of ISGs in response to viral infection of CD14+ monocytes, ARPE-19, HFFs and MoDCs was performed at 24 hpi in infected cells pre-treated with DIDS (50 uM) or DMSO control for 1 h. Data from a single experiment was analyzed in duplicate.

Previously, we have demonstrated that pre-treatment of HFFs with DIDS greatly reduced the innate immune response triggered by gB binding [28,46,47]. This was originally explained in HFFs by data demonstrating that DIDS prevented the functional interaction of HCMV with the plasma membrane. However, given that the impact of DIDS on HCMV infection of myelo-monocytic cells was post-entry, we reasoned that the activation of innate immunity could still occur. Thus, to test this, we measured the induction of classical ISGs in response to HCMV infection in CD14+ monocytes at 6 and 24 hpi. Interestingly, the induction of CXCL10, IFIT2 and IFIT3 was impaired in the presence of DIDS, although by 24 h there was still evidence of some ISG induction (albeit reduced) in the infected monocytes (Figure 7A,B). Next, we broadened our analysis to a panel of ISGs in multiple cell types. Again, the data demonstrates that DIDS broadly blocks HCMV-dependent ISG induction in all cell types tested except in ARPE-19 cells—HCMV infection did not appear to induce any major ISG induction under these experimental conditions. It was notable, in the absence of DIDS, that in myeloid cells the ISG induction was much higher than that observed in HFFs, but still DIDS reduced the induction of multiple ISGs in response to HCMV infection, including monocytes (Figure 7C). It was also evident that cell-type-specific differences were evident, whereby different ISGs were induced, and, furthermore, DIDS would have potentially differential effects depending on which cell type the ISG was being analyzed in. For example, IF44L was induced in multiple cell types but only in CD14+ monocytes; no effect was observed in the presence of DIDS, including monocyte-derived DCs (Figure 7C). Thus, within the broad failure to induce the normal programme of ISGs by viral infection, there was evidence of clear cell type-specific differences, with the caveat that the ISG array analyses would require specific qPCR follow-up for validation.

### 3.6. DIDS Has a Differential Impact on HCMV-Induced Events During the Early Stages of Myeloid Cell Infection

**Figure 8:**
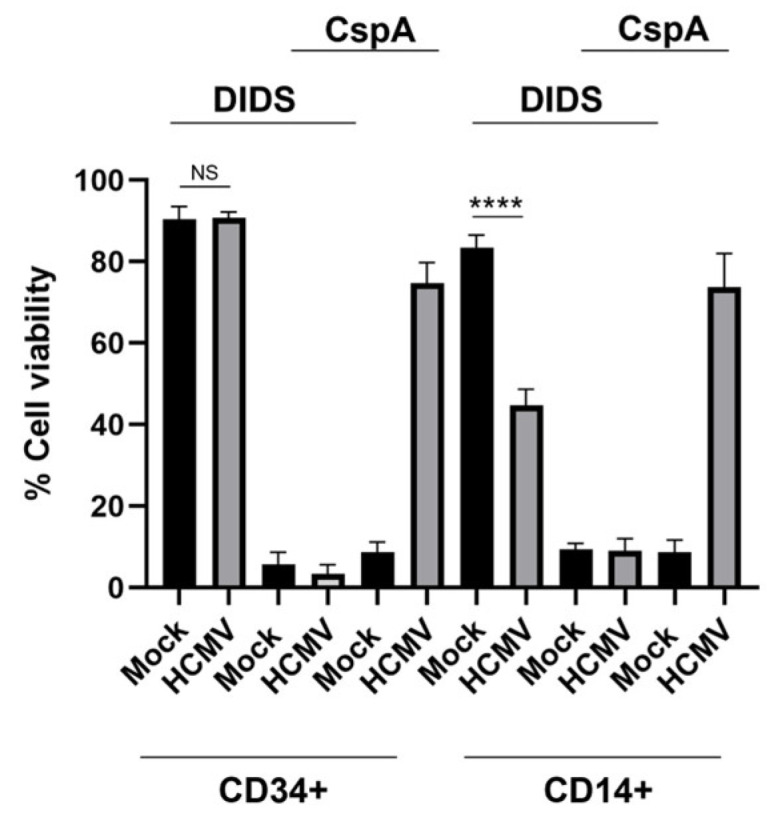
**DIDS blocks HCMV-induced cell death and survival signals**. CD34+ cells or CD14+ monocytes pre-treated with DIDS (50 uM) or DMSO for 1 h were infected with HCMV and then incubated with either DMSO or cisplatin A (CspA; 10 uM) and cell viability was assessed 24 h later by trypan blue viability and expressed relative to untreated infected controls. *n* = 3 and error bars represent 1 standard deviation from the mean. One-way ANOVA with Sidak’s multiple comparisons was performed. The difference between the CD34+ and CD14+ phenotype in DIDS is shown. **** *p* < 0.0001; NS = non-significant.

The identification of this monocyte-specific DIDS phenotype led us to investigate whether we could use this to interrogate reported phenotypes, reasoning that this differential phenotype of DIDS in myelo-monocytic infection could allow us to further interrogate events that occur upon viral binding and entry, potentially important for the establishment of latent infection. We and others have previously reported that HCMV promotes cell survival via the activation of host cell signalling pathways [35,48,49,50,51]. Furthermore, it is clear that this is necessary because there is concomitant activation of cell death pathways by viral infection (which was revealed by inhibition of the putative survival pathways). First, we infected CD34+ progenitor cells in the presence or absence of DIDS and then, 3 hpi, challenged the cells with cisplatin A or solvent control (Figure 8). The data show that DIDS completely abrogates the ability of HCMV to protect CD34+ from cisplatin A-induced cell death (Figure 8). Similarly, DIDS abrogated the ability of HCMV to protect CD14+ cells from cisplatin A-induced cell death (Figure 8). Importantly, viral infection of non-permissive cells is a trigger of cell death, which is revealed when the concomitant survival signal is blocked. However, HCMV infection of CD34+ cells in the presence of DIDS resulted in no detrimental impact on viability (Figure 8). In contrast, the infection of CD14+ cells in the presence of DIDS revealed a more complicated phenotype. Specifically, infection in the presence of DIDS was observed to induce some cell death in CD14+ cells (Figure 8), although not to the levels seen with cisplatin A (Figure 8), but potentially could explain the small loss in genomes seen earlier (Figure 5D).

**Figure 9:**
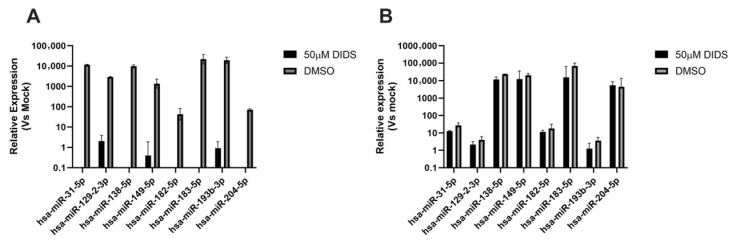
**DIDS inhibits the induction of host miRNA expression in CD34+ cells but not CD14+ monocytes.** (**A**,**B**) CD34+ (**A**) or CD14+ monocytes (**B**) were pre-treated with DIDS (50 uM) or DMSO control for 1 h, then infected with HCMV. RNA harvested at 6 hpi was then analyzed for expression of a subset of host miRNAs by qPCR and expressed relative to baseline control (or qPCR cutoff if miRNA expression not detected in mock cells) harvested prior to infection. *n* = 2 and error bars represent 1 standard deviation from the mean.

Finally, we assessed whether DIDS had any impact on the expression of host miRNAs that we have previously reported to be induced upon infection of myeloid progenitor cells [36]. An analysis of the most highly expressed miRNAs revealed that DIDS completely abrogated the upregulation of miRNAs by HCMV upon the infection of CD34+ cells (Figure 9A). In contrast, the up-regulation of this subset of miRNAs upon infection of CD14+ monocytes was not affected (Figure 9B), unlike observations in CD34+ cells (Figure 9A), suggesting that the viral binding and entry process was a sufficient trigger for their expression in CD14+ cells.

## 4. Discussion

We have previously demonstrated that DIDS is inhibitory to infection of HFFs and that inhibition is linked to cysteine reactivity in DIDS that promotes direct binding to the HCMV virion [28]. We now extend this to show that DIDS is inhibitory in multiple cell types permissive for HCMV infection, and the phenotype is broadly similar—virus entry is restricted at the plasma membrane in astrocyte-like, epithelial-like and haemotopoietic progenitors. However, in myelo-monocytic lineage committed cells, including differentiated DCs, evidence of intracellular DNA was observed. From a biological point of view, these genomes were non-functional—they were not transcriptionally active for latent gene expression, nor could lytic gene expression be induced using known activators of HCMV reactivation in THP1 cells or monocytes.

Given the capacity of DIDS to bind to virions, it is not surprising that it can block entry of HCMV into multiple cell types. However, DIDS was also active against HCMV cell-associated spread, which could be explained by multiple hypotheses that are not necessarily mutually exclusive. For example, DIDS was observed to be active against the fusogenic activity of gB in an in vitro assay, suggesting it can bind gB. DIDs binding to gB would be consistent with the viral binding and inhibition of entry data, as well as activity against cell-to-cell spread. Multiple glycoproteins at the plasma membrane are important for cell-associated spread of HCMV. It may be that DIDS directly inhibits interactions between gB and other glycoprotein complexes required to activate gB [8]. It was interesting that, in comparison, that DIDS failed to inhibit the activity of the Spike fusion protein which is potentially consistent with the idea DIDS inhibits an interaction of gB with another glycoprotein complex required for activation of gB. We cannot rule out the possibility that DIDS may be incorporated into mature virions in the replicating cell, which have been treated with DIDS. That said, DIDS carries a negative charge that suggests it is poorly taken up by cells and likely explains its lack of toxicity at very high doses both in vitro and in vivo [52].

An initially surprising observation was that, despite the uptake of HCMV by the myelo-monocytic cells in the presence of DIDS, this was not sufficient to trigger the same innate immune response as observed in normal HCMV infection. HCMV was presumably not engaging with cell surface or endosomal TLRs, which are well established to be activated upon HCMV entry [46,47,53,54]. Therefore, DIDS promoted virion entry in a largely immunologically silent way (at least from an innate immune response point of view) or, alternatively, the predominant route of entry is different in the presence of DIDS. For example, the lack of endosomal TLR activation may most likely be explained by the assumption that the virion remains intact in the endosome and never reveals the nucleic acid PAMPs [55]—which would be consistent with the transcriptional data and lack of responsiveness to reactivation stimuli. However, it also suggests that HCMV can enter (or be taken up by) these cells without strong activation of plasma membrane TLRs—although whether this is important remains unclear. Indeed, it may be that DIDS is blocking the normal routes of both membrane fusion and endosomal/macropinocytotic pathways utilized during infection of most cell types. It is also a possibility that in monocytes and DCs, virus uptake occurs additionally due to phagocytosis occurring when other pathways are blocked, or HCMV is being captured by a pathway insensitive to the activity of DIDS. For example, DC-SIGN activity on the surface of DCs is a well-established capture mechanism for multiple viruses, including HCMV [10,56], and possibly these pathways of uptake are responsible for some of the ISG gene expression seen in monocytes in the presence of DIDS. Additionally, although HCMV infection of monocytes is via an endosomal pathway, it has been shown that, in monocytes, this is a highly protracted and unique process compared to that observed in most cell types [23,57].

Elegant work from Yurochko and colleagues has also provided extensive evidence that activation of the src family kinase signalling at the plasma membrane is important for trafficking away from recycling endosomes during normal entry [23]. However, given the destructive nature of this route, this would not necessarily correlate with the long-term persistence of viral genomes observed. That said, a minor effect of DIDS on this binding interaction may contribute to the partial loss of genomes observed in the infected CD14+ cells and thus suggest that some signalling/binding events are still occurring in the presence of DIDS. More fundamentally, these different observations would be congruent with the clearly quite unique interaction of HCMV with CD14+ monocytes (and monocytic cell lines) upon entry [58,59,60,61]. Furthermore, when considering ‘latent infection’, understanding where similarities and differences between infection of short-lived CD14+ cells compared to long-lived CD34+ cells occur will be important for understanding primary infection, dissemination and pathogenesis (CD14+ monocytes) and establishment of long-term latency (CD34+ cells).

In contrast to the impaired activation of the innate immune signalling pathway upon viral infection, there appeared to be no impact on the up-regulation of a subset of miRNAs in CD14+ monocytes, which we previously identified to be up-regulated in CD34+ cells [36]—and an event DIDS blocked in CD34+ cells. Therefore, DIDS was blocking many signalling events associated with viral infection but not the upregulation of miRNAs. This differential makes sense if DIDS prevents binding of HCMV to CD34+ cells but not CD14+ cells. This is also consistent with the observation that an endocytosis inhibitor blocked the induction of miRNA expression in CD34+ cells, suggesting that the process of entry was important. Taken in the context of the CD14+ data here, it strongly suggests viral uptake (rather than binding) is the major trigger for these miRNAs to be up-regulated. It also suggests that the induction of miRNAs is partly separated from innate immune signalling activation. Thus, one intriguing hypothesis is that these up-regulated miRNAs are a host response to infection triggered by host-directed pathogen internalization that could be part of an anti-viral response [62]. Teasing this out may help determine whether the observed miRNA induction is associated with pro-viral or anti-viral responses during HCMV infection and entry into non-permissive myeloid cells.

The DIDS cell death data are also consistent with the hypothesis that specific interactions of the virus with the host cell are not only crucial for cell survival but also for the induction of cell death [50]. In the early stages of infection of CD34+ cells, protection from cell death is gB-dependent [35], and thus our observations that DIDS blocks gB function would explain why DIDS renders infected cells now sensitive to cisplatin A. However, it is clear that infection in the presence of DIDS did not kill CD34+ cells, which suggests HCMV engages with specific host functions resulting in the induction of cell death, which are also likely to be viral glycoprotein-driven. This is perhaps easily explained by the fact that DIDS appeared to inhibit HCMV at the plasma membrane in CD34+—so neither the survival nor death signal was initiated—and, subsequently, may also explain the more nuanced monocyte phenotype. Although DIDS again prevented HCMV-mediated protection from cisplatin A cell death, some monocyte cell death was observed even in the presence of DIDS. Thus, either a death signal was being engaged to some extent in these cells, and one that is DIDS insensitive (and thus potentially another glycoprotein complex except gB), or, alternatively, because CD14+ monocytes are inherently short-lived, the loss of the survival signal was sufficient to see evidence of loss of cell viability [63]. Again, we are reminded of the work from Yurochko, Chan and colleagues demonstrating that the short-lived nature of CD14+ monocytes (and thus an increased propensity for spontaneous death) renders them dependent on a persistent survival signal via Pi3K [48,64], which involves sustained gB signalling via EGFR [65]. These data would suggest the hypothesis that DIDS, which reduces gB fusogenic activity in vitro, could diminish this critical interaction as well.

## 5. Conclusions

In summary, we present data that reports on the further characterization of DIDS as an anti-viral compound that inhibits HCMV infection, both cell-free and cell-associated. In the process of characterizing the anti-viral activity of DIDS, we identify an intriguing differential phenotype in myelo-monocytic cells whereby infection is inhibited post-entry but only triggers limited events associated with viral infection. Thus, DIDS has the potential to be used to further dissect the sequence of interactions of HCMV with the host cell. Specifically, we consider that DIDS may prove useful as a tool to identify and dissect events that are important for and during virus entry and, in turn, mechanisms underlying important components of the host response aimed at limiting viral infection.

## Data Availability

The raw data supporting the conclusions of this article will be made available by the authors on request.
